# A Stability-Indicating Ultra Performance Liquid Chromato-Graphic (UPLC) Method for the Determination of a Mycophenolic Acid-Curcumin Conjugate and Its Applications to Chemical Kinetic Studies

**DOI:** 10.3390/molecules26175398

**Published:** 2021-09-05

**Authors:** Yonelian Yuyun, Ponsiree Jithavech, Worathat Thitikornpong, Opa Vajragupta, Pornchai Rojsitthisak

**Affiliations:** 1 Biomedicinal Chemistry Program, Department of Biochemistry and Microbiology, Faculty of Pharmaceutical Sciences, Chulalongkorn University, Bangkok 10330, Thailand; yoneli_redrose@yahoo.com; 2 Natural Products for Ageing and Chronic Diseases Research Unit, Chulalongkorn University, Bangkok 10330, Thailand; ponsireejithavech@yahoo.com; 3 Department of Food and Pharmaceutical Chemistry, Faculty of Pharmaceutical Sciences, Chulalongkorn University, 254 Phayathai Road, Pathumwan, Bangkok 10330, Thailand; worathat.t@pharm.chula.ac.th; 4 Research Affairs, Faculty of Pharmaceutical Sciences, Chulalongkorn University, Bangkok 10330, Thailand; opa.v@chula.ac.th

**Keywords:** stability-indicating assay, UPLC, chemical kinetics, curcumin, mycophenolic acid, prodrug

## Abstract

A simple, precise, and accurate reversed-phase ultra-performance liquid chromatographic (UPLC) method was developed and validated for the determination of a mycophenolic acid-curcumin (MPA-CUR) conjugate in buffer solutions. Chromatographic separation was performed on a C18 column (2.1 × 50 mm id, 1.7 µm) with a gradient elution system of water and acetonitrile, each containing 0.1% formic acid, at a flow rate of 0.6 mL/min. The column temperature was controlled at 33 °C. The compounds were detected simultaneously at the maximum wavelengths of mycophenolic acid (MPA), 254 nm, and curcumin (CUR), or MPA-CUR, at 420 nm. The developed method was validated according to the ICH Q2(R1) guidelines. The linear calibration curves of the assay ranged from 0.10 to 25 μg/mL (r^2^ ≥ 0.995, 1/x^2^ weighting factor), with a limit of detection and a limit of quantitation of 0.04 and 0.10 μg/mL, respectively. The accuracy and precision of the developed method were 98.4–101.6%, with %CV < 2.53%. The main impurities from the specificity test were found to be MPA and CUR. Other validation parameters, including robustness and solution stability, were acceptable under the validation criteria. Forced degradation studies were conducted under hydrolytic (acidic and alkaline), oxidative, thermal, and photolytic stress conditions. MPA-CUR was well separated from MPA, CUR, and other unknown degradation products. The validated method was successfully applied in chemical kinetic studies of MPA-CUR in different buffer solutions.

## 1. Introduction

**Figure 1:**
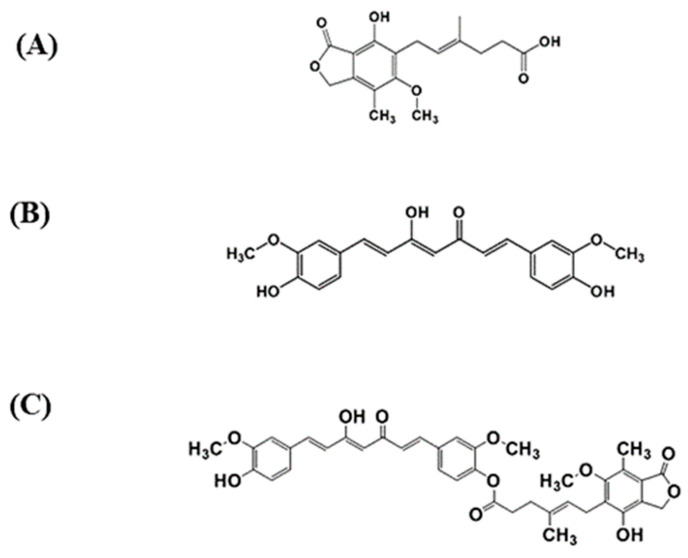
The chemical structures of mycophenolic acid (MPA, **A**), curcumin (CUR, **B**), and mycophenolic acid-curcumin (MPA-CUR, **C**).

A mutual prodrug strategy derives several benefits from having two active compounds in one molecule [1]. We recently synthesized a novel mycophenolic acid-curcumin (MPA-CUR) conjugate as a prodrug of mycophenolic acid (MPA) and curcumin (CUR) (Figure 1) [2]. The bioavailable fraction (BF) of MPA-CUR across Caco-2 cells showed better anti-psoriatic and anti-inflammatory effects than that of CUR in a TNF-α-induced HaCaT cell model [2], suggesting MPA-CUR as a potential candidate for psoriasis treatment [2]. Because MPA-CUR requires bioconversion, giving parent molecules to exert anti-psoriatic activity, chemical kinetic studies of MPA-CUR are useful in determining the stability of MPA-CUR under physiological conditions. Prodrug stability is usually carried out in buffer solutions representing physiological environments, such as the stomach (pH 1–2), the intestine (pH 5.5–7) and blood (pH 7.4) [3]. In addition, the released amount of the parent molecule from its prodrug provided essential information for the dose regimen design [4,5]. Accordingly, a stability-indicating assay (SIA) for the determination of MPA-CUR in physiological buffers is required for chemical kinetic studies, as part of a physicochemical property determination that can serve as initial guidance for the prediction of the pharmacokinetics of drug candidates.

The obligation to establish an SIA has become more clearly specified since the International Conference on Harmonisation (ICH) and the introduction of the U.S. Food Drug Administration (FDA) guidelines [6]. According to the U.S. FDA, all testing methods for chemical stability studies must demonstrate a stability-indicating character. Chromatographic approaches are often applied in this regard due to the specificity of the technique. Sample preparation, method development, and method validation are the three components required to implement SIA. Stress testing, also known as forced degradation, can be performed in order to offer knowledge regarding drug degradation processes that may arise during storage. It also aids with formulation development, fabrication, and packaging. Forced degradation studies must be conducted under several conditions, including pH, light, oxidation, moisture, and heat, that can prove the separation of the drug from its degradants, according to the guidelines [6,7]. 

The determination of CUR has been studied using several methods in various dosage forms, biological samples, and derivative substances [8]. In prior studies, liquid chromatography was used to analyze CUR and its ester prodrugs [9,10]. The combination of high-performance liquid chromatography with UV-Vis spectroscopy (HPLC-UV) was the first analytical technique established and applied in the investigation of the chemical kinetics of curcuminoid prodrugs [9]. Other HPLC-UV methods have been developed to determine curcumin ester prodrugs and released CUR in plasma or Caco-2 permeates [10,11,12]. Several methods for determining MPA or its ester prodrug in bulk [13], dosage form [13,14,15], and biological samples [16] have been published. An MPA assay in a biological matrix is described in most articles for therapeutic drug monitoring using HPLC or UPLC-MS [16]. Chopade et al. published a validated HPLC-based analytical technique for MPA in MPA- aminosugar prodrugs using a C18 column with a mobile phase consisting of a phosphate buffer at pH 4.5 and acetonitrile [17]. However, the SIA has not been proven using the analytical method developed by Chopade et al. [17]. To date, the simultaneous determination of CUR and MPA has not been reported. 

In the present study, a simple, accurate, precise, and specific ultra-performance liquid chromatography (UPLC) method for the quantitative determination of MPA-CUR in buffer solutions was developed and validated. Forced degradation studies were conducted under hydrolytic (acidic and alkaline), oxidative, thermal, and photolytic stress conditions to demonstrate the specificity of the method. The validated method was successfully applied in chemical kinetic studies of MPA-CUR in different buffer solutions.

## 2. Results and Discussion

### 2.1. Method Development

**Figure 2:**
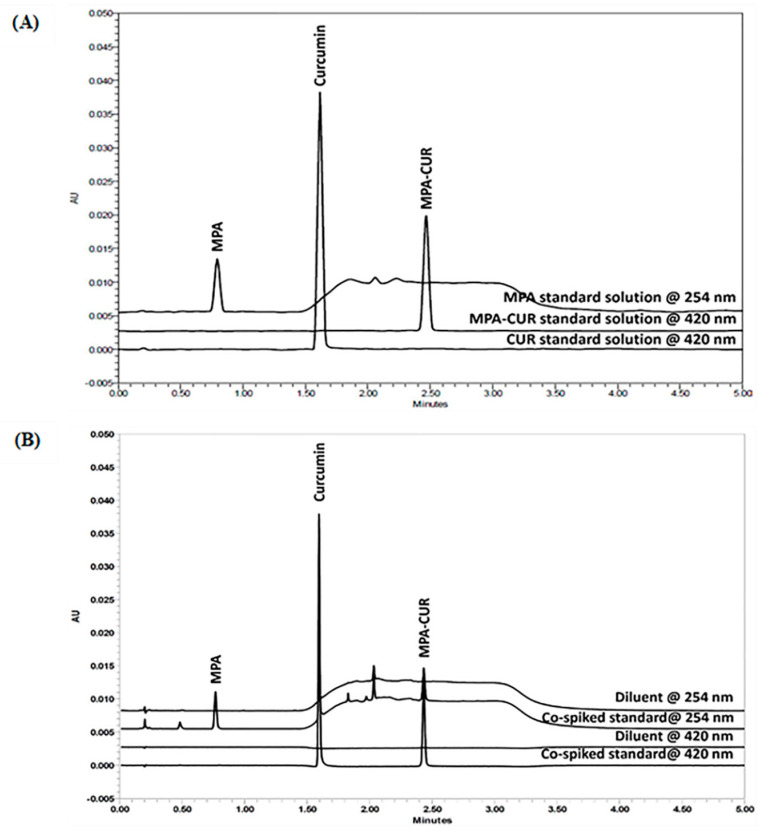
(**A**) The overlaid chromatograms of MPA standard solution at 254 nm, and CUR and MPA-CUR standard solutions at 420 nm. (**B**) The representative overlaid chromatograms of co-spiked MPA, CUR, and MPA-CUR at 254, 420 and 420 nm, respectively, and diluents at 254 nm and 420 nm.

The UPLC analytical method was developed to quantify MPA-CUR in the samples used in the chemical kinetic study, which is an important topic in the physicochemical analysis of new substances. The MPA-CUR stability in various buffer solutions was investigated. CUR and MPA are the starting materials for the MPA-CUR conjugate and can be potential hydrolytic products. Therefore, the developed method for the quantitative estimation of MPA-CUR required the efficient separation of MPA-CUR from CUR, MPA, and unknown degradation products. The chromatographic separation of MPA-CUR from its degradation products was achieved with the gradient elution program, varying the ratio of 0.1% *v*/*v* formic acid in water (A) and 0.1% *v*/*v* formic acid in acetonitrile (B) at a flow rate of 0.6 mL/min. The acetonitrile was gradient-eluted for 2.7 min to ensure that the analytes could be separated on the column (from 0–2.7 min). The MPA was eluted in 0.8 min (Figure 2A), corresponding to a 40% acetonitrile elution. For the elution of CUR and MPA-CUR, the acetonitrile was kept at 70% from 1.0–2.5 min. The CUR and MPA-CUR took 1.6 and 2.5 min, respectively, to elute in 70% of acetonitrile. Initially, HPLC was used for the analysis and the mobile phase was optimized. Water and acetonitrile, containing 0.1% formic acid each, were found to be suitable as aqueous and organic phases, respectively, to separate MPA, CUR, and MPA-CUR. However, the total analysis time of 30 min was too long, and therefore UPLC was used in this study, providing the shorter total analysis time of 5 min. Acetonitrile is a preferred reverse-phase liquid chromatography organic modifier because of its physicochemical properties, such as the increased strength of its reverse-phase eluent, its lower viscosity, and its shorter wavelength UV cut-off [18,19]. Our experiment used formic acid at 0.1% *v*/*v* as a modifier due to its increased sensitivity and preserved analyte peaks that were sharp and symmetrical, as previously published [20,21]. The advantage of adding 0.1% *v*/*v* formic acid to water and acetonitrile is that it causes a constant concentration of 0.1 %*v*/*v* formic acid in the mobile phase during the gradient analyzer. Due to the molecular weight of MPA-CUR, we used the C18 column in our study, which is suitable for non-polar molecules [12]. An Acquity UPLC™ BEH C18 column (2.1 × 50 mm, 1.7 µm) was adopted to provide good separation and tolerate the low pH of the mobile phase. The UPLC pressure during the elution with a flow rate of 0.6 mL/min was about 6700 pounds per square inch (psi). The UPLC used in this study was equipped with binary pumps, which can tolerate up to 15,000 psi [22,23]. The spectral analysis report provided by the photodiode array detector was used to analyze the chromatographic peak purity data based on the maximum wavelengths of MPA, at 254 nm, and CUR, or MPA-CUR, at 420 nm. The developed UPLC method was efficient in separating MPA-CUR from its degradation products in the forced degradation samples.

### 2.2. Method Validation

#### 2.2.1. System Suitability

A system suitability test was performed to determine the reproducibility of the used system. The reproducibility of the method was expressed using the percentage deviation (%CV) from the retention time and the peak area for five injections. The resolution between CUR and MPA-CUR was 29, which is greater than 2. The %CV values of the retention time and the peak area of the MPA-CUR conjugate were found to be 0.14% and 0.28%, respectively. Based on the average value of the USP tailings of the MPA-CUR conjugate, it can be concluded that this method provided adequate chromatographic performance, with an asymmetrical peak shape with a value of 1.0. The number of theoretical plates was more than 2000. The chromatogram of the system suitability result is provided in Figure 2. The summarized data from the suitability testing in Table 1 met the limit criteria requirements, and the chromatographic conditions were suitable for the MPA-CUR analysis. 

#### 2.2.2. Specificity and Forced Degradation Study

Specificity plays an important role in the separation capability of methods developed between analytes in the presence of disturbances such as solvents, matrices, and potential impurities [12]. The specificity results shown in Figure 2B indicated that the chromatographic conditions could separate MPA, CUR, and MPA-CUR. The baseline drift at 254 nm and the same retention time of CUR and MPA-CUR did not impact the peak response of CUR and MPA-CUR due to the use of different detection wavelengths. Furthermore, no interference by the diluent in the retention times of MPA, CUR and MPA-CUR was observed (Figure 2B).

**Figure 3:**
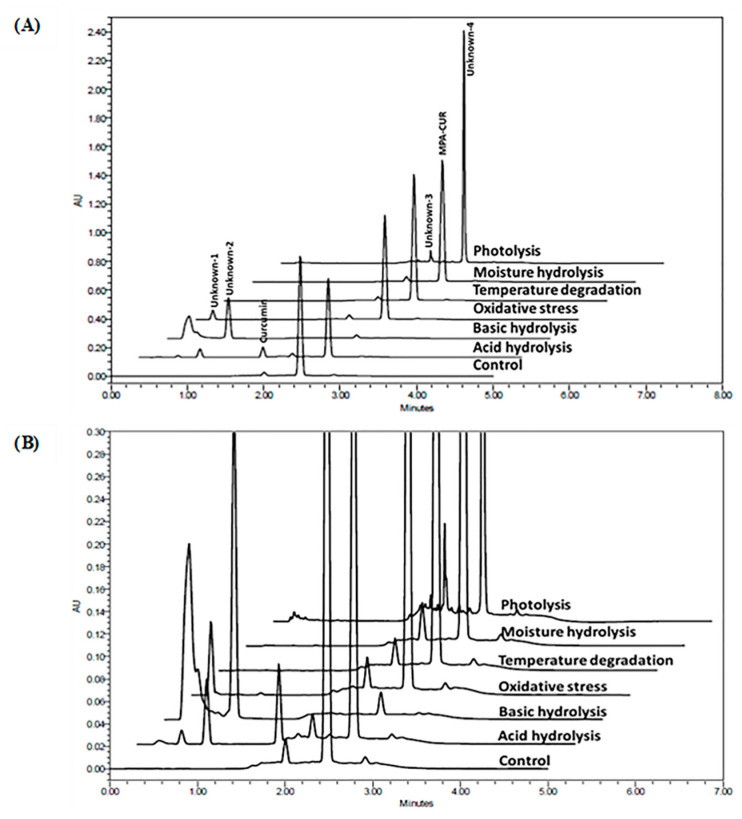
(**A**) The overlaid chromatograms for MPA-CUR exposed to various forced degradation conditions. (**B**) The overlaid chromatograms (extended scale) for MPA-CUR exposed to different forced degradation conditions.

The forced degradation study represents the possible degradation products under various stress factors and, subsequently, provides information on the mechanism of degradation. Specificity is the ability of a method to distinguish target compounds from their impurities [24]. The results of the forced degradation study are given in Table 2. The specificity of the developed method was determined by the peak purity and the MPA-CUR remaining after the forced degradation study via a photodiode array detector. The peak purity was considered from the relationship between the purity threshold and the peak purity angle. It was considered that there was no coelution if the peak of interest had a purity threshold greater than the purity angle. According to Table 2, the results showed that the proposed method could separate MPA-CUR from other degradants in all stress conditions. The representative overlaid chromatograms from the forced degradation experiments at 420 nm are demonstrated in Figure 3. The results suggested that MPA-CUR is unstable when exposed to acid, base, and light. In acidic and basic stress conditions, the main degradation pathway is through ester hydrolysis, which releases the MPA and CUR detected. Under basic stress conditions, MPA-CUR was found to be much more unstable than under other stress conditions. The MPA-CUR was almost lost in the remaining samples. The fact that it was undetectable under the wavelength of 420 nm is due to the instability of CUR in the basic buffer. CUR is highly degradable to other compounds [25]. Interestingly, MPA-CUR is relatively unstable under photo-stress conditions, according to the ICH Q1B guidelines [26]. We observed that, after being exposed for not less than 1.2 million lux hours near a UV and fluorescent lamp, MPA-CUR could mostly degrade into an unknown impurity at a retention time of 2.38 min (Figure 3B), which can be separated from the MPA-CUR with a resolution of 3 under the chromatographic conditions we developed. 

#### 2.2.3. Linearity and Range

**Figure 4:**
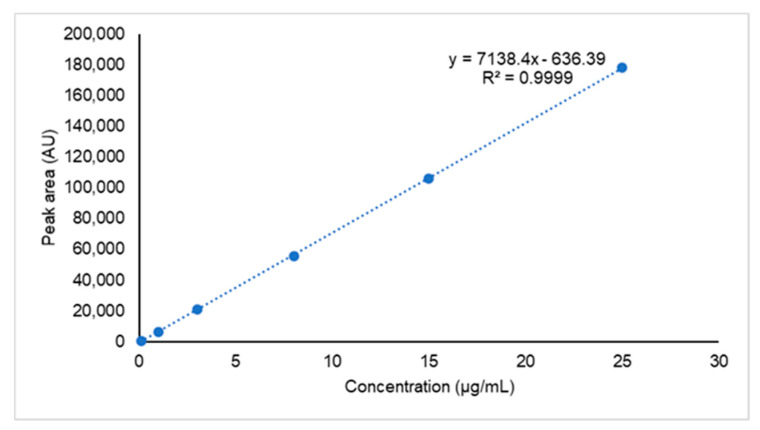
Linear calibration curve of UPLC analysis of MPA-CUR.

The correlation coefficient (r) and the coefficient of determination (r^2^) were used to assess the quality (strength) of the regression [27]. The representative visualization of the calibration curve with the coefficient of determination (r^2^) of 0.9999 is shown in Figure 4. Several statistical tests have been proposed to determine the linearity of the calibration range, including the lack-of-fit (LOF) test [28]. The linearity of the calibration curve was confirmed through the LOF test, which gave *F*_(*cal)*_ < *F*_(*tab*)_, indicating that the ordinary least square model is appropriate to fit the data.

Since the data range was extensive, with more than one order of magnitude, an unweighted linear regression for calibration curve experiments with heteroscedasticity might have resulted in inaccurate analysis results, especially at lower concentration ranges [29,30]. A homoscedasticity test was also carried out in a concentration range of 0.1–15 mg/L [29]. The regression line and homoscedasticity of the calibration curve were assessed in order to establish whether the ordinary or the weighted least square were adequate for the robust calibration model [31]. 

The homoscedasticity test was performed and the results are presented in Table 3. The highest and lowest concentrations in the calibration curve data were used to calculate an *F* value to determine if there was a significant difference in the variances of the two groups [32,33]. The *F*_(*cal*)_ value found was 219,113.642, which was significantly higher than *F*_(*table*)_ = 99.000 (*F_2,2,0.99_*). Therefore, the weighted linear least square was applied for the generation of the linear equation in this experiment.

The three-replicate calibration line was evaluated using the weighted linear square model with a weighting factor, as presented in Table 4. The weighted regression model with a weighting factor of 1/x^2^ had the lowest %RE and was ideal for homogenizing residual variance, indicating that 1/x^2^ was the best weighting factor. A weighting value of 1/x^2^ was applied to calculate the calibration range of 0.10 to 25 μg/mL. The calibration curve of MPA-CUR was linear in the range of 0.10–25 µg/mL, with a high correlative of determination (r^2^ = 0.997). The slope and intercept values for the first replication were found at 7003.813 and −61.143, respectively, with the second and third replication being close to this value. 

The selected linearity equation was then tested using back-calculated concentration data, and is presented in Table 5. The percentage of the relative error of the mean back-calculated concentration and actual concentrations of MPA-CUR were in the range of −5.10 and 1.81. The %CV (*n* = 3) of the back-calculated concentration was less than 4.40. The residual plots and regression analysis were generated using one-way analysis of variance in order to demonstrate that the *F* values (*F_table_*) of all the regression lines were significantly less than the calculated *F* value (*F*_cal_). The results are presented in Table 5. They indicated a good linear relationship between the peak response (y) and the analyte concentration (x). The *p*-value was the regression parameter used to indicate whether the slope and y-intercept were significantly different from zero at a 95% confidence interval. The *p*-values of the slope and y-intercept were also calculated, as summarized in Table 5. The results demonstrated that the *p*-values of the slope were less than 0.05, indicating a significant difference from zero, while the *p*-values of the intercept exceeded 0.05, indicating that the intercepts of all the regression lines were insignificantly different from zero. Therefore, the calibration standard curve can be applied for the routine analysis of MPA-CUR, and a single-point calibration standard can be used for the single-point assay.

#### 2.2.4. Limit of Detection (LOD) and Limit of Quantification (LOQ)

The LOD and the LOQ were predicted based on signal-to-noise by determining the signal of low MPA-CUR concentrations compared to the signal of the diluent (noise). As shown in Table 6, the LOD of the MPA-CUR conjugate was 0.04 µg/mL, with an S/N ratio of 3. The LOQ of the MPA-CUR conjugate was 0.10 µg/mL, with an S/N ratio of 12. Regarding the LOQ, the accuracy and precision were represented as %recovery in the range of 90.5 to 94.1%, with a %CV of 1.6, respectively. All the results indicated a satisfactory method sensitivity for MPA-CUR analysis.

#### 2.2.5. Accuracy and Precision

The Intra- and inter-day accuracy and precision were evaluated at three levels of spiked samples, including 0.10 (LOQ), 12.5, and 25 µg/mL in triplicate (*n* = 3). The results are summarized in Table 7. All of the spiked quality control (QC) samples for intra-day accuracy exhibited a %recovery in a range from 98.4 to 101.6%, with a %CV < 0.81. Regarding the inter-day evaluation, three-day accuracy demonstrated %recovery in the range from 98.5 to 101.2 with %CV < 2.53. The accuracy and precision results showed the good accuracy and precision of the proposed method.

#### 2.2.6. Robustness

For the method robustness assessment, we performed suitability testing using five injections with variations of the formic acid concentration in the mobile phase and the column. The concentration of the formic acid solution was varied in the range of ± 0.01% from the proposed method condition. In addition, the batch-to-batch variation of the analytical column was evaluated from two different batches of analytical columns. As shown in Table 8, there was no effect on the system’s reproducibility, represented as %CV of retention time and peak area of MPA-CUR (%CV < 0.84), due to the slight variation of formic acid concentration and the different column batches. In addition, the system’s performance expressed as the tailing factor (T < 1.1) and the number of theoretical plates (N = 12,587) was not affected by the variation, indicating that the proposed method is robust under the above variations. 

#### 2.2.7. Stability of the MPA-CUR Solutions in the Autosampler

To ensure the stability of the sample solution during analysis, the MPA-CUR was studied by incubating the sample solution in the autosampler at 37 °C and sampling at different time intervals. The %recovery from the initial sample solution was 99.66–103.23% (Table 9). The data indicated that the sample solution was relatively stable up to 24 h in an autosampler at 37 °C. An autosampler set at 37 °C can be used for chemical kinetic studies.

### 2.3. Application of the MPA-CUR Determination in Chemical Kinetic Studies

**Figure 5:**
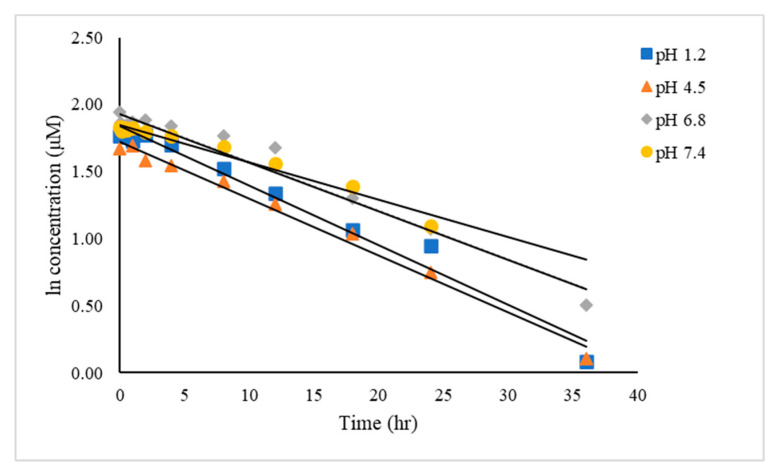
The pseudo-first-order degradation plots of MPA-CUR in buffer solutions of pH 1.2, 4.5, 6.8, and 7.4.

The chemical kinetic for the MPA-CUR conjugate in different buffer solutions, including pH 1.2, 4.5, 6.8 and 7.4 under 37 °C, was determined using a validated method at a detection wavelength of 420 nm in order to monitor the remaining MPA-CUR. The natural logarithmic plots of the MPA-CUR concentration in buffers versus time were linear for all conditions tested, as presented in Figure 5, indicating that the degradation of MPA-CUR followed pseudo-first-order kinetics. The order kinetic of reaction can be determined using various methods, including the graphic method based on r^2^ value [34]. The kinetic model that showed the highest r^2^ value was selected to determine kinetic parameters. The results, presented in Table 10, showed that the pseudo-first-order model was suitable for the determination of the chemical stability of MPA-CUR. The overall degradation rate constants (*k_obs_*) and half-life (*t*_1/2_) of MPA-CUR in buffer pH 1.2, 4.5, 6.8, and 7.4 are shown in Table 11. The MPA-CUR was stable in all pH conditions tested. The half-lives (*t*_1/2_) of MPA-CUR ranged from 15.67 to 19.73 h in buffer pH conditions tested. CUR was previously found to degrade rapidly at pH 7.4 with *t*_1/2_ of 0.56 h [10], indicating that MPA-CUR seems more stable than CUR. The conjugation between MPA and CUR delayed the release of MPA or CUR themselves, a process that might enable the molecule to become gradual across a cell membrane.

## 3. Materials and Methods

### 3.1. Chemicals and Reagents

The MPA (M.W. 320.3 g/mol) was obtained from AK Scientific (Union City, CA, USA). The CUR and MPA-CUR (purity > 98% by HPLC) were prepared and characterized in our laboratory using the previously published method [2,9]. The analytical grades of formic acid and dimethyl sulfoxide (DMSO) were bought from Carlo Erba (Parc d’affaire des Portes, Val de Reuil, France). The HPLC grade of acetonitrile and methanol was purchased from Fisher Scientific (Loughborough, Leicester, UK). The reagent-grade glacial acetic acid, potassium chloride, and monobasic potassium phosphate were obtained from Scharlab (Sentmenat, Barcelona, Spain). The quinine monohydrochloride dihydrate USP standard (Lot no. R071S0, purity 100%) was purchased from USP. The ultrapure water was obtained using a Milli-Q^®^ integral water purification system (Milli-Q, MA, USA). The hydrogen peroxide and sodium hydroxide were obtained from Carlo Erba (Sabadell, Barcelona, Spain). The hydrochloric acid (37% *w*/*v*) was purchased from QRëc (Auckland, New Zealand).

### 3.2. Chromatographic Conditions

The chromatographic instrument used was the Acquity UPLC™ system (Waters Corporation, Milford, MA, USA), equipped with an autosampler, photodiode array detector, quaternary solvent manager, and column oven compartment. The data collection and analysis were performed on Waters Empower 3 software (Waters Corporation, Milford, MA, USA). The injection volume was 1 µL, and the chromatographic separation was obtained on an Acquity UPLC™ BEH C18 column (2.1 × 50 mm, 1.7 µm). The mobile phase consisted of 0.1% formic acid in water (A) and 0.1% formic acid in acetonitrile (B), with gradient elution at a flow rate of 0.6 mL/min. The gradient elution program was optimized as follows: an initial of A-B 60:40 at 0.0 min; an isocratic gradient of A-B 60:40 from 0.0–0.9 min; a linear gradient of A-B 30:70 from 0.9–1.0 min; an isocratic gradient of A-B 30:70 from 1.0–2.5 min; a linear gradient of A-B 60:40 from 2.5–2.7 min; and an isocratic gradient of A-B 60:40 from 2.7–5.0 min. The column and autosampler temperature were set at 33 °C and 15 °C, respectively. The detection wavelengths were set at 254 nm for MPA and 420 nm for CUR and MPA-CUR. 

### 3.3. Preparation of the Standard Solution 

A stock standard solution of MPA-CUR (100 µg/mL) was prepared by dissolving 2 mg of MPA-CUR with 20 mL of dimethyl sulfoxide (DMSO) in a 20-mL volumetric. Accurately, a 0.8 mL of MPA-CUR stock solution (100 µg/mL) was transferred into a 10-mL volumetric flask and diluted with acetonitrile to obtain MPA-CUR at a concentration of 8 µg/mL. The standard stock solutions of MPA and CUR were prepared in the same manner. Subsequently, all standard solutions were filtered through 0.22 μm nylon membrane filters before analysis.

### 3.4. System Suitability

For the system suitability test, the solution was prepared by diluting the standard stock solutions of CUR, MPA, and MPA-CUR (100 μg/mL) with the diluent to obtain a solution containing 8 μg/mL of each compound. The solution was passed through a nylon filter of 0.22-µm pore size prior to analysis.

### 3.5. Forced Degradation Studies

The forced degradation studies were performed in order to confirm that the developed method of analysis had specificity in the separation of MPA-CUR from its degradation end products. The synthesized MPA-CUR conjugate was treated under the various stress conditions recommended in the regulatory guidelines [6]. The control and stressed sample solutions were analyzed using UPLC coupled with a photodiode array detector (PDA) to verify the peak purity of the remaining MPA-CUR peak. The chromatogram of the force degradation study was presented at a wavelength of 420 nm.

#### 3.5.1. A Control Sample

A 15-mL glass-stopper test tube was filled with 1 mg of MPA-CUR, followed by 100 μL of water. The mixture was vortexed until it was completely homogenous. The sample was dissolved with 10 mL of DMSO. The obtained solution was then transferred to a 50-mL volumetric flask and diluted with the DMSO to volume. Subsequently, 1 mL of this solution was transferred into a 10-mL volumetric flask and diluted to volume with the acetonitrile.

#### 3.5.2. Acid Hydrolysis

A 15-mL glass-stopper test tube was filled with 1 mg of MPA-CUR, followed by 100 μL of 0.1 N HCl. The mixture was vortexed until it was completely homogenous. For 3 h, the sample was maintained at 80 °C. The remaining acid was then neutralized with 100 μL of 0.1 N NaOH. The sample was dissolved with 10 mL of DMSO. The obtained solution was then transferred to a 50-mL volumetric flask and diluted with the DMSO to volume. Subsequently, 1 mL of this solution was transferred into a 10-mL volumetric flask and diluted to volume with the acetonitrile.

#### 3.5.3. Basic Hydrolysis

A 15-mL glass-stopper test tube was filled with 1 mg of MPA-CUR, followed by 100 μL of 0.1 N NaOH. The mixture was vortexed until it was completely homogenous. For 3 h, the sample was maintained at 80 °C. The remaining acid was then neutralized with 100 μL of 0.1 N HCl. The sample was dissolved with 10 mL of DMSO. The obtained solution was then transferred to a 50-mL volumetric flask and diluted with the DMSO to volume. Subsequently, 1 mL of this solution was transferred into a 10-mL volumetric flask and diluted to volume with the acetonitrile.

#### 3.5.4. Moisture Hydrolysis

In a 15-mL glass-stopper test tube, 1 mg of MPA-CUR was dispersed in 100 μL water. The mixture was vortexed until it was completely homogenous. The sample was heated for 3 and 6 h at 80 °C. After that, the leftover sample was dissolved with 10 mL of DMSO. The obtained solution was then transferred to a 50-mL volumetric flask and diluted with the DMSO to volume. Subsequently, 1 mL of this solution was transferred into a 10-mL volumetric flask and diluted to volume with the acetonitrile.

#### 3.5.5. Oxidative Degradation

In a 15-mL glass-stopper test tube, 1 mg of MPA-CUR was treated with 100 μL of 3% H_2_O_2_ and incubated at room temperature and 80 °C for 1 h. Next, the residual sample was dissolved in 10 mL DMSO and then transferred to a 50-mL volumetric flask. The DMSO was used to dilute the transferred solution to volume. Subsequently, 1 mL of the solution was pipetted into a 10-mL volumetric flask and diluted to volume with the diluent.

#### 3.5.6. Thermal Degradation

In a 15-mL glass-stopper test tube, 1 mg of MPA-CUR was heated at 80 °C for 3 and 6 h. The sample was then dissolved in 10 mL DMSO, transferred to a 50-mL volumetric flask, and volume-adjusted with the DMSO. A 1 mL of the solution was then transferred to a 10-mL volumetric flask and diluted with acetonitrile to the final volume.

#### 3.5.7. Photolysis

The photostability of MPA-CUR was examined in solid form, according to the ICH Q1B guidelines [26]. The light intensity indicator was a 2% *w*/*v* aqueous solution of quinine monohydrochloride dihydrate in a 1-cm quartz cell. During the investigation, the quinine solution was placed next to the test sample. Next, 1 mg of MPA-CUR was placed in a photostability chamber at room temperature with direct exposure to fluorescence and UV light and left for five days, until a change of at least 0.5 was observed in the UV absorbance of the quinine solution at 400 nm. The tested sample was then dissolved in 10 mL of DMSO. The obtained solution was then transferred to a 50-mL volumetric flask and diluted with DMSO to volume. Subsequently, 1 mL of this solution was transferred into a 10-mL volumetric flask and diluted to volume with the acetonitrile.

### 3.6. Method Validation

In terms of assay techniques, the method was validated according to the ICH Q2(R1) guidelines for the validation of analytical procedures [6].

#### 3.6.1. System Suitability 

A system suitability test is a prerequisite for the performance evaluation of a chromatographic system before the beginning of the analysis. The MPA-CUR solution at a concentration of 8 µg/mL was used for the system suitability test. The system’s repeatability was evaluated under the five-injection repeatability via coefficient variation (%CV) of the retention time and peak area. In addition, the system’s performance was assessed under column efficiency via tailing factor (T) and theoretical plate (*N*). The %CV of the five replicates injection should be less than 2%, while the tailing factor (T) should be less than 2. In addition, the number of the theoretical plate (*N*) should be greater than 2000 [35,36]. 

To verify the system’s performance, a fresh system suitability solution containing a mixture of CUR, MPA, and MPA-CUR at a concentration of 8 μg/mL for each compound was generated. The CUR and MPA-CUR resolutions were determined. Since MPA has a different maximum wavelength to MPA-CUR, the resolution between MPA and MPA-CUR was not examined.

#### 3.6.2. Specificity 

CUR and MPA compounds could be potential degradation products of MPA-CUR in different conditions, such as various buffer pH, oxidation, moisture, temperature and light. The system suitability samples and forced degradation samples were prepared. The specificity was determined by separate injections of the diluent, the MPA-CUR standard solution (8 μg/mL), the CUR standard solution (8 μg/mL), the MPA standard solution (8 μg/mL), the mixture of standard solutions of CUR, MPA and MPA-CUR, and the forced degradation samples.

#### 3.6.3. Linearity and Range

Calibration standard solutions were prepared by diluting appropriate volumes of the standard stock solution of MPA-CUR (100 μg/mL) in acetonitrile. A series of concentrations for linearity was prepared in the range of 0.10–25 µg/mL. The MPA-CUR standard solution was evaluated at 0.10, 1, 3, 8, 15 and 25 µg/mL. The calibration curve with three replicates was constructed by plotting the peak area of MPA-CUR as a function of the concentrations. The linearity for the calibration curve was evaluated by applying the lack-of-fit (LOF) test. The homoscedasticity was tested to assess whether the weighted linear regression model was needed [37]. In the case of no homoscedasticity, a weighted-linear least square model with a weighting factor would be applied. The weighted linear calibration model with the lowest percentage of relative error (RE) was chosen as the best calibration model [32]. The coefficient of determination (r^2^) should be greater than 0.995. The slope and intercept were also determined. The equation’s suitability was confirmed using a back-calculation of the calibration standard concentration. The %relative error (%RE) of the back-calculation regression line can be used to express deviations from the proposed linear calibration model. The acceptable %RE is ± 20 to the limit of quantification (LOQ) and ± 15 to the rest of the nominal concentration [28,38]. The regression analysis of the residual plot is often used for the determination of whether the slope and y-intercept are significantly different from zero at a 95% confidence interval. In the linear calibration method, the slope must be statistically different from 0, and the intercept must not be statistically different from 0 by statistical calculations [39]. The linear relationship between the peak response (y) and the concentration (x) can also be assessed from the *F*_value_ when *F*_cal_ is greater than *F*_ANOVA_ [40].

#### 3.6.4. LOD and LOQ 

The LOD and the LOQ are indicators of a method’s sensitivity [36,41]. The stock standard solution of MPA-CUR (100 μg/mL) was diluted to obtain a LOD solution at a concentration of 0.04 μg/mL. The LOD is accepted if the signal-to-noise ratio of the analyte response is greater than 3, while the precision of injection (*n* = 5) at the LOD must provide the precision of injection with a %CV of lower than 15 [12,42]. LOQ solution was obtained by diluting stock standard solutions of MPA-CUR (100 μg/mL) to obtain the final concentration at 0.10 μg/mL. The LOQ is accepted if the signal-to-noise ratio of the analyte response is greater than 10. In contrast, the analyte response (*n* = 5) at this concentration must provide a %recovery in the range of 80–110%, with a %CV of lower than 15 [12,42].

#### 3.6.5. Accuracy and Precision

The quality control (QC) samples were prepared by diluting the stock standard solutions (100 μg/mL) to obtain the final concentrations at 0.10 (LOQ), 12.5, and 25 µg/mL in triplicate (*n* = 3). The triplicates of three QC samples of the MPA-CUR were used to determine intra-day accuracy and precision. Meanwhile, three replicates of the MPA-CUR QC were tested in triplicate on three different days for inter-day accuracy and precision. The accuracy was assessed via the calculation of %recovery. The percentage of recovery should be in the range of 80–110% [42]. The precision was evaluated via the percentage of coefficient variation (%CV) by dividing the standard deviation by the concentration mean. The %CV should be ≤7.3 for all concentrations, excluding the LOQ, which should be ≤15 [42].

#### 3.6.6. Robustness

The method’s robustness determines whether the system’s suitability remains unaffected by small changes in the method parameters. Five injections of MPA-CUR solutions at 8 μg/mL were prepared for robustness testing by diluting the stock standard solution of MPA-CUR (100 μg/mL). The analytical procedure was evaluated by a slight variation of the method parameters, including the percentage of formic acid content (±0.01%) from an original chromatographic condition of 0.1% formic acid. In addition, batch-to-batch variation was evaluated using two different batches of the analytical column. The unbiased results were assessed through the system suitability parameters, as mentioned in Section 3.6.1, to ensure the efficiency of the proposed method under small variations.

#### 3.6.7. Stability of the MPA-CUR Solutions in the Autosampler

The stability of the working standard solutions of MPA-CUR was studied under a controlled temperature to ensure the stability of the sample solution during incubation. The autosampler temperature was set at 37 °C, which was used for further chemical kinetics studies. The 8 μg/mL sample solution was prepared in the same manner as mentioned in Section 3.3 for the stability test. The sample solution was kept in a thermostat autosampler with a temperature of 37 °C for 24 h. The %Recovery of MPA-CUR content from the initial time was calculated at 6, 9,12, and 24 h after incubation and should be in the range of 80–110% [42]. 

### 3.7. Application of the MPA-CUR Determination for Chemical Kinetic Studies

For chemical kinetic studies, the validated method was applied to the determined MPA-CUR in buffer solutions at pH 1.2, 4.5, 6.8 and 7.4. Stock solutions of MPA-CUR at 100 µg/mL were prepared, as mentioned in Section 3.3. The system’s suitability and calibration standards in the range of 0.10–25 μg/mL were prepared using the stock solution (100 μg/mL). The samples of the chemical kinetic studies were prepared by adding 50 µL of the stock solution (100 µg/mL) to 950 µL of each medium, i.e., 0.1 M HCl (pH 1.2), 0.1 M acetate buffer pH 4.5, and phosphate buffer (pH 6.8 and 7.4), to give a final concentration of 5 µg/mL. The solution was left to stand in the thermostat autosampler of the UPLC instrument at a temperature of 37 ± 0.1 °C for 24 h. The remaining amount of MPA-CUR was determined at different time intervals. The studies were performed in triplicate. Kinetic parameters (*k_obs_* and *t*_1/2_) were determined by a natural logarithmic plot of concentration against time and calculated by linear least-squares regression analysis.

## 4. Conclusions

An accurate and reproducible, stability-indicating UPLC method was developed for the quantitative analysis of MPA-CUR. The suggested method is the first stability-indicating method to be developed and applied in the stability assay of MPA-CUR. All method validation procedures followed the ICH Q2(R1) guidelines, and the validation results showed good specificity, linearity, accuracy, precision, and robustness. The forced degradation study demonstrated that the MPA-CUR conjugate was highly labile to basic hydrolysis and photolysis. The drug remained stable in moisture, oxidative, and thermal stress conditions. The MPA-CUR was slightly labile in acid hydrolysis. The validated method was further applied in the determination of MPA-CUR in kinetic stability studies in buffer solutions. Furthermore, the proposed method here can be employed for the quality control of MPA-CUR raw materials and dosage forms.

## Figures and Tables

**Table 1:** System suitability data (*n* = 5).

Injection No.	Retention Time (min)	Peak Area	USP Tailing Factor	USP Plate Count
1	2.423	56,048	1	13,002
2	2.430	56,025	1	12,925
3	2.429	55,648	1	12,866
4	2.428	55,929	1	12,824
5	2.423	55,887	1	12,973
Mean	2.427	55,907	1	12,918
%CV	0.14	0.28	0.00	0.57

**Table 2:** Forced degradation for MPA-CUR.

Stress Condition	Incubation Time (h)	Purity Angle	Purity Threshold	MPA-CUR Remaining (%)	Peak Purity
Control (untreated)	0	0.190	0.435	100.00	Passed
Acid hydrolysis (100 µL of 0.1 N HCl), 80 °C	3	0.086	0.462	77.65	Passed
Basic hydrolysis (100 µL of 0.1 N NaOH), 80 °C	3	0.025	12.054	3.21	Passed
Oxidation (100 µL of 3% H_2_O_2_)	0	0.130	0.393	94.88	Passed
Oxidation (100 µL of 3% H_2_O_2_), 80 °C	1	0.152	0.373	91.16	Passed
Moisture hydrolysis (100 µL of water), 80 °C	3	0.197	0.409	96.85	Passed
Moisture hydrolysis (100 µL of water), 80 °C	6	0.196	0.440	99.60	Passed
Temperature degradation, 80 °C	3	0.212	0.475	102.20	Passed
Temperature degradation, 80 °C	6	0.222	0.452	101.20	Passed
Photolysis (UV and Fluorescence)	5 days	1.077	5.290	0.03	Passed

**Table 3:** The homoscedasticity test.

Standard (μg/mL)	Peak Area		Peak Area Ratio	s^2^	F_cal_	F_table_
0.100	643	1.000	643	11.533	219,113.642	99.000
	620	1.000	620			
	633	1.000	633			
24.942	177,307	1.000	177,307	5398		
	173,466	1.000	173,466			
	184,125	1.000	184,125			

**Table 4:** The weighted least-squares regression analysis for MPA-CUR (*n* = 3).

Replication	Model	Weighting Factor	Slope	Intercept	r	r^2^	∑|%RE|	Minimum	Result
1	1	1	7096.149	−112.938	0.9999	0.9990	23.727	20.43	1/*x*^2^
	2	1/*x*	7093.347	−107.486	0.9998	0.9997	22.790		
	3	1/*x*^2^	7003.813	−61.143	0.9985	0.9970	20.430		
2	1	1	6973.427	−441.224	0.9999	0.9990	57.482	10.59	1/*x*^2^
	2	1/*x*	6928.311	−123.907	0.9999	0.9998	17.914		
	3	1/*x*^2^	6809.330	−62.322	0.9996	0.9993	10.593		
3	1	1	7368.774	−885.046	0.9999	0.9998	114.5429	13.63	1/*x*^2^
	2	1/*x*	7269.613	−173.504	0.9998	0.9996	25.753		
	3	1/*x*^2^	7084.538	−77.709	0.9994	0.9989	13.625		

**Table 5:** The mean inter-day, back-calculated standard and calibration curve results (*n* = 3).

Compound	Nominal Conc. (µg/mL)	Back-Calculated Concentration (µg/mL)			Mean Back-Calculated Concentration (µg/mL)	%RE	%CV
		Day 1	Day 2	Day 3			
**MPA-CUR**	0.100	0.101	0.100	0.100	0.100 ± 0.001	0.250	0.50
	1.000	0.898	0.953	0.945	0.949 ± 0.03	−5.100	4.40
	3.000	3.175	2.986	2.942	3.026 ± 0.124	0.858	3.39
	8.000	8.071	7.927	7.996	7.996 ± 0.072	−0.053	0.74
	15.000	15.035	15.383	15.282	15.175 ± 0.179	1.167	1.23
	25.000	25.325	25.484	26.001	25.453 ± 0.353	1.810	1.64
	r^2^	0.9997					
	*F*_cal_	52,968.10878					
	*F*_table_	1.35816 × 10^−29^					
	*p*-value of slope	1.35816 × 10^−29^					
	*p*-value of intercept	0.1596					

**Table 6:** The limit of detection (LOD) and the limit of quantitation (LOQ) (*n* = 5).

Injection No.	LOD		Sample No.	LOQ			
	Peak Area	S/N		Added Conc. (µg/mL)	Found Conc. (µg/mL)	%Recovery	S/N
1	178	4	1	0.100	0.094	93.9	9
2	171	3	2	0.100	0.091	90.8	11
3	184	3	3	0.100	0.091	90.5	11
4	193	3	4	0.100	0.094	94.1	14
5	191	4	5	0.100	0.093	93.3	14
Mean	183	3			0.093	92.5	12
%CV	5.0				1.6		

**Table 7:** The accuracy and precision of the method.

Nominal Conc. (µg/mL)	Intra-Day (*n* = 3)				Inter-Day (*n* = 9)			
	Added Conc. (µg/mL)	Found Conc. (µg/mL)	%Recovery	%CV	Added Conc. (µg/mL)	Found Conc. (µg/mL)	%Recovery	%CV
0.10	0.100	0.098 ± 0.001	98.4	0.52	0.10	0.099 ± 0.001	98.5	0.77
12.5	12.47	12.71 ± 0.01	101.6	0.04	12.47	12.64 ± 0.16	101.2	1.23
25.0	24.942	25.15 ± 0.20	100.6	0.81	24.942	24.96 ± 0.63	99.8	2.53

**Table 8:** The robustness of the method (*n* = 5).

Chromatographic Parameters	Retention Time	Peak Area	Tailing Factor (T)	Theoretical Plate (*N*)
	%CV	%CV		
Concentrations of formic acid solution				
0.09%	0.08	0.84	1.0	12,615
0.10%	0.23	0.44	1.0	13,182
0.11%	0.13	0.51	1.0	12,587
Analytical columns from different batches				
Column # 1 Batch no. 0293370651	0.23	0.44	1.0	13,182
Column # 2 Batch no. 0318381361	0.08	0.59	1.1	12,609

**Table 9:** The stability of the MPA-CUR solutions at 8 µg/mL in an autosampler set at 37 °C (*n* = 1).

Time (h)	Added Conc. (µg/mL)	Found Conc. (µg/mL)	%Recovery
0	7.922	7.894	99.66
6	7.922	8.146	102.83
9	7.922	8.099	102.24
12	7.922	8.092	102.15
24	7.922	8.177	103.23

**Table 10:** The kinetic equation for chemical kinetic studies of MPA-CUR in buffer solutions at various pH at 37 °C.

pH of Buffer Solutions	r^2^		
	Zero Order	Pseudo-First Order	Second Order
1.2	0.964	0.978	0.862
4.5	0.949	0.982	0.934
6.8	0.966	0.972	0.883
7.4	0.964	0.977	0.804

**Table 11:** The kinetic parameters for chemical kinetic studies of MPA-CUR in buffer solutions at various pH at 37 °C (*n* = 3).

pH of Buffer Solutions	Kinetic Parameters	
	*k_obs_* (h^−1^)	*t*_1/2_ (h)
1.2	0.045 ± 0.003	15.67 ± 1.2
4.5	0.041 ± 0.010	18.59 ± 7.5
6.8	0.036 ± 0.036	19.73 ± 4.4
7.4	0.044 ± 0.002	15.94 ± 0.8

## Data Availability

Not available.

## Sample Availability

Not available.
